# P-1686. Appropriateness of Antibiotic Prescribing in Urgent Care Centers in Chicago, 2019-2022

**DOI:** 10.1093/ofid/ofae631.1852

**Published:** 2025-01-29

**Authors:** Linda F Li, Estrella Cervantes, Kelly Walblay, Stephanie R Black, Do Young Kim

**Affiliations:** Chicago Department of Public Health, Chicago, Illinois; The Chicago Department of Public Health, Chicago, Illinois; Chicago Department of Public Health, Chicago, Illinois; Chicago Department of Public Health, Chicago, Illinois; Chicago Department of Public Health, Chicago, Illinois

## Abstract

**Background:**

Urgent care centers (UCC) face unique challenges such as high patient volume and turnover, contributing to increased overall antibiotic prescriptions and inappropriate prescribing. The Chicago Department of Public Health (CDPH) obtained the IQVIA Longitudinal Prescription and Medical Claims dataset to evaluate antibiotic prescribing trends between 2019-2022 in Chicago UCC, including appropriateness.

**Methods:**

We analyzed IQVIA data from 2019-2022, restricted to encounters with Chicago UCC providers. Claims data were linked to prescription data using a unique patient identifier. Using previously published methodology, antibiotic appropriateness was assessed by assigning ICD-10 codes to three tiers: tier 1 conditions, for which antibiotics are almost always indicated; tier 2, antibiotics may be indicated; and tier 3, antibiotics are almost never indicated.

**Results:**

There were 24,776 antibiotic prescriptions with matched diagnosis codes attributed to Chicago UCC providers between 2019-2022, representing 19,809 unique patients. Tier 3 conditions accounted for 44.6% of prescriptions, followed by tier 2 (37.0%) and tier 1 (18.4%) conditions . The most common tier 3 diagnosis codes were respiratory infections (43.2%), including viral upper respiratory infections (40.4%), bronchitis/bronchiolitis (11.9%), and other respiratory conditions (37.7%). Inappropriate prescribing was lowest in 2019 and highest in 2021, with 40.1% and 48.3% of prescriptions matched to a tier 3 condition, respectively. The three most frequently prescribed antibiotics following an encounter for a tier 3 condition were azithromycin (25.2%), amoxicillin (12.3%), and amoxicillin/clavulanate (11.4%). Nurse practitioners (29.1%) were the most frequent tier 3 prescribers followed by family medicine (25.4%) and internal medicine (24.0%) physicians.

**Conclusion:**

Nearly half of antibiotics prescribed in Chicago UCC from 2019-2022 were associated with a condition where antibiotics are almost never indicated, and this trend increased during the COVID-19 pandemic. Antibiotic stewardship efforts should target unnecessary antibiotic treatment for respiratory infections, and stewardship education should prioritize nurse practitioners, and family medicine and internal medicine physicians.

**Disclosures:**

**All Authors**: No reported disclosures

